# Long Non-Coding *PROX1-AS1* Expression Correlates with Renal Cell Carcinoma Metastasis and Aggressiveness

**DOI:** 10.3390/ncrna7020025

**Published:** 2021-04-10

**Authors:** Magdalena Rudzinska, Karolina H. Czarnecka-Chrebelska, Ekaterina B. Kuznetsova, Sofya V. Maryanchik, Alessandro Parodi, Dmitry O. Korolev, Nataliya Potoldykova, Yulia Svetikova, Andrey Z. Vinarov, Marina V. Nemtsova, Andrey A. Zamyatnin

**Affiliations:** 1 Institute of Molecular Medicine, Sechenov First Moscow State Medical University, 119991 Moscow, Russia; kuznetsova.k@bk.ru (E.B.K.); mar97son@yandex.ru (S.V.M.); aparodi.sechenovuniversity@gmail.com (A.P.); nemtsova_m_v@mail.ru (M.V.N.); 2 Department of Biomedicine and Genetics, Medical University of Lodz, 90-419 Lodz, Poland; karolina.czarnecka@umed.lodz.pl; 3 Laboratory of Epigenetics, Research Centre for Medical Genetics, Moskvorechye str. 1, 115478 Moscow, Russia; 4 Institute for Urology and Reproductive Health, Sechenov University, 119992 Moscow, Russia; demix84@inbox.ru (D.O.K.); potoldykova_n_v@staff.sechenov.ru (N.P.); ukukushkina92@gmail.com (Y.S.); avinarov@mail.ru (A.Z.V.); 5 Belozersky Institute of Physico-Chemical Biology, Lomonosov Moscow State University, 119991 Moscow, Russia; 6 Department of Biotechnology, Sirius University of Science and Technology, 1 Olympic Ave, 354340 Sochi, Russia

**Keywords:** non-coding RNA, PROX1-AS1, renal cell carcinoma, human specimens

## Abstract

Long non-coding RNAs (lncRNAs) can be specifically expressed in different tissues and cancers. By controlling the gene expression at the transcriptional and translational levels, lncRNAs have been reported to be involved in tumor growth and metastasis. Recent data demonstrated that multiple lncRNAs have a crucial role in renal cell carcinoma (RCC) progression—the most common malignant urogenital tumor. In the present study, we found a trend towards increased *PROX1* antisense RNA 1 (*PROX1-AS1*) expression in RCC specimens compared to non-tumoral margins. Next, we found a positive correlation between *PROX1-AS1* expression and the occurrence of distant and lymph node metastasis, higher tumor stage (pT1 vs. pT2 vs. pT3–T4) and high-grade (G1/G2 vs. G3/G4) clear RCC. Furthermore, global demethylation in RCC-derived cell lines (769-P and A498) and human embryonic kidney 293 (HEK293) cells induced a significant increase of *PROX1-AS1* expression level, with the most remarkable change in HEK293 cells. In line with this evidence, bisulfite sequencing analysis confirmed the specific demethylation of bioinformatically selected CpG islands on the *PROX1-AS1* promoter sequence in the HEK293 cell line but not in the tumor cells. Additionally, the human specimen analysis showed the hemimethylated state of CG dinucleotides in non-tumor kidney tissues, whereas the tumor samples presented the complete, partial, or no demethylation of CpG-islands. In conclusion, our study indicated that *PROX1-AS1* could be associated with RCC progression, and further investigations may define its role as a new diagnostic marker and therapeutic target.

## 1. Introduction

Renal cell carcinoma (RCC) is a deadly genitourinary malignancy characterized by metastases and chemotherapy resistance [1]. The major RCC histological variants are clear cell renal cell carcinoma (KIRC), accounting for 70% of cases, papillary renal cell carcinoma (KIRP) ~10–15%, and chromophobe cell carcinoma (KICH) ~5% and others ~15% [2,3]. Tyrosine kinase inhibitors, such as sunitinib, axitinib, and pazopanib, inhibiting the vascular endothelial growth factor receptor cascade, represent the first-line treatment for this disease. Unfortunately, RCC eventually develops resistance to these medications, so novel therapeutic strategies and approaches are urgently required [4]. Understanding RCC molecular basis is paramount for discovering new therapeutic interventions against this disease [1,5].

Long non-coding RNAs (lncRNAs) represent a group of molecules with a length of over 200 nucleotides that do not code for proteins, despite having a similar structure to functional mRNA [6]. LncRNAs display tissue-specific expression patterns and have been shown to affect a broad range of biological functions [7]. LncRNAs have initially been considered to regulate gene expression at the post-transcriptional level, but a growing body of evidence indicates they also play an important role in epigenetic control [8,9]. On the other hand, lncRNA expression can be regulated by epigenetic mechanisms, including methylation [10].

It was already shown that lncRNAs play a vital role in the development of renal diseases, including fibrosis [11], autosomal dominant polycystic kidney disease [12], diabetic kidney disease [13], and RCC [14,15]. Recent data indicated that a series of lncRNA covers a regulatory function and may have a prognostic value in RCC (Table 1).

In this scenario, understanding lncRNA biology in kidney cancer disease may provide new diagnostic and therapeutic opportunities.

The most common renal cancer metastatic sites are the lungs (45%), followed by the bones (30%) and lymph nodes (22%) [22]. It was estimated that RCC tumor lymph node infiltration is associated with distant metastases occurrence, significantly reducing patient 5-year survival [23]. In this context, one of the primary lymphatic markers—Prospero homeobox 1 (*PROX1*)—was detected in RCC-derived cells as a factor correlated with elevated tumor aggressiveness and lymph node invasion [24].

**Figure 1:**
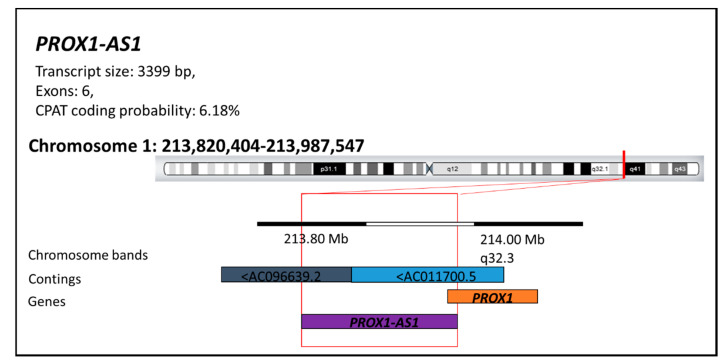
The *PROX1* antisense RNA 1 structure (*PROX1-AS1*). The *PROX1-AS1* presents a transcript of 3399 bp, including six exons with a faint (6.18%) probability of protein translation (according to the Coding-Potential Assessment Tool (CPAT)). LncRNA-*PROX1-AS1* is located on the long arm of chromosome 1 at position 1q32.3 (start position: 213820404; end position: 213987547) in the regions AC096639.2-AC011700.5 of the DNA segments. This information is available at LNCipedia [29], RNA central [30], and Ensemble [31] databases.

Both *PROX1* and its antisense strand—*PROX1-AS1* (3399 bp) located on human chromosome 1q32.3 (Figure 1), can be actively involved in tumor progression [25,26,27,28].

Previous studies demonstrated a significantly increased *PROX1-AS1* expression in papillary thyroid cancer, prostate, and ovarian tumor specimens compared to adjacent non-tumoral tissues. Moreover, an enhanced *PROX1-AS1* expression increased the invasion of different cancer cells in vitro [28,32,33].

Herein, we investigated and correlated RCC specimen *PROX1-AS1* expression to control tissues, distant and lymph node metastatic events, higher tumor stages, and high-grade tumors. Furthermore, the bioinformatic simulation and data derived from in vitro and ex-vivo experiments indicate that an epigenetic mechanism could be involved in *PROX1-AS1* expression.

## 2. Materials and Methods

### 2.1. Tissues Samples

The samples included in this study comprised RCC (T) and the surrounding non-cancerous kidney (NT) tissues. Fresh samples were snap-frozen in liquid nitrogen and stored at −80 °C. A partial nephrectomy was performed for localized disease, including 3–5 mm of peritumoral margin collected outside the cancer tissue and tumor capsule. All removed structures were morphologically examined. The samples derived from patients with pT1-2N0M0 renal masses treated with laparoscopic and robotic partial nephrectomy (*n* = 28 (67%)) and patients with pT3-4 N0-1 M0-1 renal masses treated with laparoscopic radical nephrectomy (*n* = 14 (33%)) at the Institute for Urology and Reproductive Health, Sechenov University, Moscow, Russia between 2019 and 2020.

The research was carried out following the ethical proceedings approved by the Ethical Committees of Sechenov University, Moscow, Russia. The samples were collected from 42 patients, including 22 males and 20 females, with a median age of 58.8 years (range 26–80 years). Seven patients developed metastasis, and three had lymph node tumor invasion. Lymph node metastasis were detected before surgery via CT scan examination. If the lymph nodes were more than 1.0–2.0 cm, lymphadenectomy followed by histopathological examination was performed. RCC sample postoperative histopathological verifications were performed according to the International Agency for Research on Cancer (2016) WHO classification of tumors of the urinary system and male genital organs (IARC WHO classification of tumors), 4th ed. Written informed consents were received from all participant patients before they participated in the study. The study was conducted in accordance with the Declaration of Helsinki, and the protocol was approved by the Ethics Committee at Sechenov University, Moscow, Russia of N04–12.

### 2.2. Cell Lines

Human 786-P, A498 RCC cell lines, and embryonic kidney cells HEK293 were kindly provided by Dr. Vadim Pokrovsky (purchased from American Type Culture Collection). The cells were grown in RPMI 1640 or DMEM/F12 medium supplemented with 10% fetal bovine serum and 1% mixture of antibiotics penicillin-streptomycin (all from Gibco, Waltham, MA, USA) at 5% CO_2_ and 37 °C in a humidified atmosphere containing 5% CO_2_. Cells were cultivated as it was previously described [34]. All tested cell lines were authenticated by STR DNA Profiling Analysis (GORDIZ, Moscow, Russia).

### 2.3. RNA Isolation and Real-Time (RT)-qPCR

Tissues were disintegrated and fragmentized using TissueLyser LT or TissueLyser II and steel beads (Qiagen, Hilden, Germany). Total RNA was extracted from kidney specimens and cultured cells using the Total RNA isolation kit (Evrogen, Moscow, Russia) according to the manufacturer’s protocol. RNA quality and concentration were evaluated using NanoDrop spectrophotometer (NanoDrop Technologies Inc., Wilmington, DE, USA). Samples with the A260/A280 ratio ~2.0 were used for further analyses and stored at −80 °C.

Complementary DNA (cDNA) was transcribed from mRNA using cDNA synthesis kit (Evrogen, Moscow, Russia), according to the manufacturer’s protocols. For RT reaction 1 µg of total RNA was used with optical density OD260/OD280 1.7–2.0 measured with NanoDrop One (Thermo Fisher Scientific, Waltham, MA, USA). Expression of the human genes was quantified by RT-qPCR using the cDNAs as a template in reactions containing the double-stranded DNA-specific dye BioMaster HS-qPCR SYBR Blue (2x) (BiolabMix, Novosibirsk, Russia) and specific oligonucleotide primers: (1) *PROX1-AS1*: F-5′-CTAGTTAGCAGGGGCAGCAC-3′, R-5′-AACAGAGAGGCGTGGAAGAA-3′; (2) *GAPDH* F-5′-AGAAGGCTGGGGCTCATTTG-3′, AGGGGCCATCCACAGTCTTC-3′. PCR reactions were performed in triplicates with the following conditions: 95 °C/30 s, 40 cycles of 95 °C/5 s, 60 °C/15 s and 72 °C/10 s in iQ5 Real-Time PCR Detection System (Bio-Rad, Hercules, CA, USA). The cycle threshold (Ct) values estimated for analyzed genes were normalized against corresponding Ct values of *GAPDH* gene. The relative quantification value (RQ) was calculated as the relative change in *PROX1-AS1* transcript expression level compared to the *PROX1-AS1* level in the internal control represented by a mixture of RNA extracted from control samples.

### 2.4. Methylation Analysis

The methylation simulation was performed in the 1q32.3 region (start position chr1: chr1: 213,985,153–end position: 214,000,000 on the *Homo sapiens* chromosome 1, GRCh38.p13 Primary Assembly) harboring the lncRNA-*PROX1-AS1* (ncRNA: NR_037850.2) exon 1 and the gene’s promoter localized 3kB upstream from the *PROX1-AS1* transcription start point. CpG sites prediction was performed using the online UCSC Genome Browser tool (http://genome.ucsc.edu; accessed on 9 April 2021).

### 2.5. In Vitro Treatment of Cultured Cells with 5-Aza-2′-Deoxycytidine

Cells were seeded at a density of 1.0 × 10^6^ cells in T25 flasks and placed at 37 °C overnight in a 5% CO_2_ incubator. The next day, the cell culture medium was replaced with a fresh medium containing 5 μM 5-aza-2′-deoxycytidine 5-AZA and cultured for the next 48 h.

### 2.6. DNA Isolation, Bisulfite Modification and Sequencing PCR

Genomic DNA from kidney cancer patients and cell cultures HEK293, 786-P, and A498 were isolated using a standard phenol-chloroform method. Bisulfite conversion was performed using the EpiTect Bisulfite kit (Qiagen, Hilden, Germany) to distinguish the methylated from non-methylated cytosines in the DNA sequence.

#### Bisulfite Sequencing PCR

The fragments of CpG- islands (1, 2, 3) were amplified from the *PROX1-AS1* promoter region, using specific primers for bisulfite sequencing PCR (Table 2). Bisulfite sequencing PCR was performed using BigDye™ Terminator v3.1 Cycle Sequencing Kit (Thermo Fisher Scientific, Waltham, MA, USA). DNA methylation status of the selected CpG islands were validated with bisulfite Sanger sequencing—on the automatic genetic analyzer 3500 (Thermo Fisher Scientific) according to the manufacturer’s protocols.

### 2.7. Statistical Analysis

Statistical analysis was performed using Statistica13.1 (StatSoft, Tulsa, OK, USA). When comparing independent groups, ANOVA Kruskal–Wallis (for comparison of two groups), U Mann–Whitney tests (for comparison of three and more groups) with additional multiple comparison (two-sided) test were used to evaluate the relationship between lncRNA expression level (RQ) and patients’ characteristics (age and sex of the patient) and clinical features of the tumor (staging according to Tumour Node Metastasis (TNM), American Joint Committee on Cancer Staging (AJCC) and histopathological RCC subtypes. Correlation of lncRNA expression in RCCs and non-cancerous kidney tissues within groups were performed using Spearman’s rank correlation. The results of relative expression analysis (RQ values) are presented as mean ± SD for normal distribution. *p* < 0.05 was considered statistically significant.

## 3. Results

### 3.1. LncRNA PROX1-AS1 Expression Is Higher in RCC Specimens and Positively Correlates with Metastasis, Lymph Node Invasion, Tumor Stage, and Grade

To evaluate the prognostic role of PROX1-AS1 in RCC progression, we investigated its expression in 42 RCC (T) samples and the corresponding surrounding non-cancerous kidney tissues (NT) (Table 3).

*PROX1-AS1* expression analysis showed an increase in T compared to paired NT tissues in the case of KIRC and KICH groups and a decrease in KIRP and renal angiomyolipoma (AML). However, the revealed differences were found to be not statistically significant. A positive correlation between *PROX1-AS1* expression in T and NT was notified for all studied groups, containing KIRC, KICH, KIRP, and AML patients (Spearman’s rank correlation; R = 0.307, *p* < 0.05). Additionally, a positive correlation of *PROX1-AS1* relative expression was observed in KIRC patients with metastasis (M1) between T and NT (Spearman’s rank correlation; R = 0.48, *p* < 0.05). Interestingly in women, the *PROX1-AS1* level was higher in T than in NT, while in men, it was the opposite; however, changes were determined to be not statistically significant.

**Figure 2:**
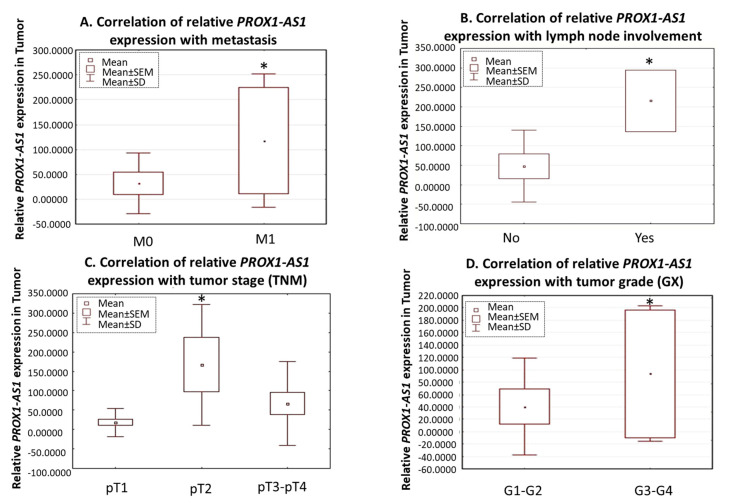
Expression of *PROX1-AS1* in human renal cell carcinomas. (**A**) *PROX1-AS1* expression is higher in patients with metastases (M1) compared to patients without metastasis (M0). (**B**) Increased *PROX1-AS1* expression corresponded to lymph node tumor infiltration. (**C**) *PROX1-AS1* increased in the higher tumor stages (pT1 vs. pT2 and pT3–4). (**D**) *PROX1-AS1* significantly increased in G3–G4 vs. G1–G2 grades in clear renal cell carcinomas. * *p* < 0.05.

The correlation with the clinicopathological data showed a remarkably higher relative expression of *PROX1-AS1* in patients with distant metastasis (U Mann-Whitney test, *p* = 0.0435; Figure 2A) and lymph node invasion (U Mann-Whitney test, *p* = 0.04; Figure 2B). We also observed that *PROX-AS1* expression increased in pT2-pT4 compared to pT1. *PROX-AS1* expression was significantly elevated in pT2 (compared to pT1, *p* = 0.03), but not in pT3-4 (Figure 2C), showing the typical bell-shaped distribution of *PROX1-AS1* expression for tumor stage. Furthermore, among KIRC patients, *PROX1-AS1* expression in T positively correlated with the higher grades G3/G4 compared to G1/G2 (Wilcoxon pair order test, *p* = 0.04, Figure 2D).

### 3.2. Methylation Could Regulate PROX1-AS1 Expression in Healthy Kidney and Renal Carcinoma Cells

**Figure 3:**
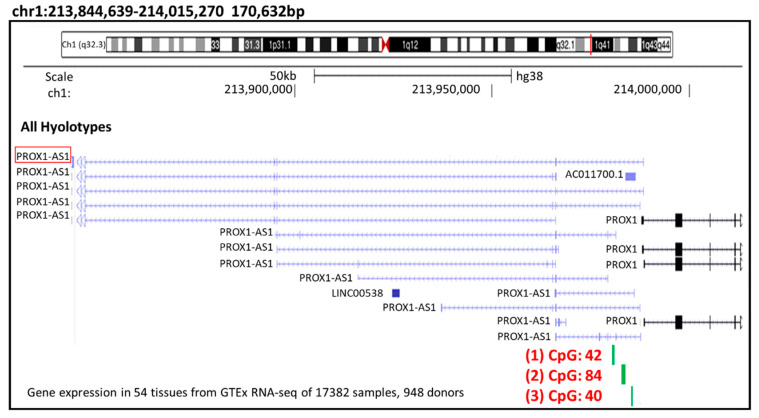
*PROX1-AS1* promoter region CpG island analysis. In the methylation simulation performed in the lncRNA-*PROX1-AS1* exon 1 and the gene’s promoter (start position chr1: 213,985,153–end position: 214,000,000, accession data: >NC_000001.11:213985153-214000000 *Homo sapiens* chromosome 1, GRCh38.p13 Primary Assembly), three CpG islands (1: CpG 42; 2: CpG 84 and 3: CpG 40) were selected with UCSC Genome Browser (http://genome-euro.ucsc.edu/; accessed on 9 April 2021).

To investigate the possible relationship between the methylation and lncRNA *PROX1-AS1* expression we performed a bioinformatic analysis of the lncRNA *PROX1-AS1* promotor region, searching for CpG islands (Figure 3).

In the methylation simulation conducted in the lncRNA-*PROX1-AS1* exon 1 and the gene’s promoter (localized 3 kB upstream from the *PROX1-AS1* transcription start point) using UCSC Genome Browser (http://genome-euro.ucsc.edu/; accessed on 9 April 2021), three CpG islands located in the *PROX1-AS1* promoter sequence were selected for further analysis (Table 4).

**Figure 4:**
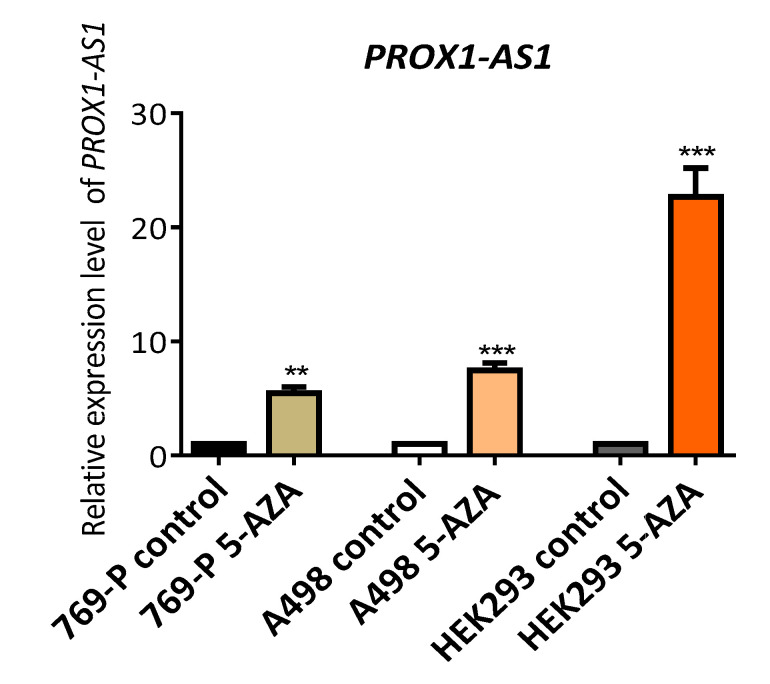
The demethylation induced by 5-aza-2′-deoxycytidine increased the expression of *PROX1-AS1* in human renal carcinoma cells (769-P and A498) and embryonic kidney 293 cells (HEK293). *PROX1-AS1* expression was determined by qRT-PCR following exposure of cells with 5 uM 5-aza-2′-deoxycytidine for 48 h. ** *p* ≤ 0.01, *** *p* ≤ 0.001.

The treatment of human RCC-derived (769-P and A498) and embryonic kidney cells (HEK293) with the demethylating agent 5-aza-2′-deoxycytidine resulted in a notable increase of *PROX1-AS1* expression level (Figure 4) with the most significant change in HEK293 cells. These results indicated that methylation could contribute to *PROX1-AS1* expression regulation in renal carcinomas and non-tumor kidney cells.

After bisulfite sequencing of the selected CpG-islands, 1 (CpG 42), 2 (CpG 84), and 3 (CpG 40), the presence of cytosine hypermethylation was observed (Table 5.).

**Figure 5:**
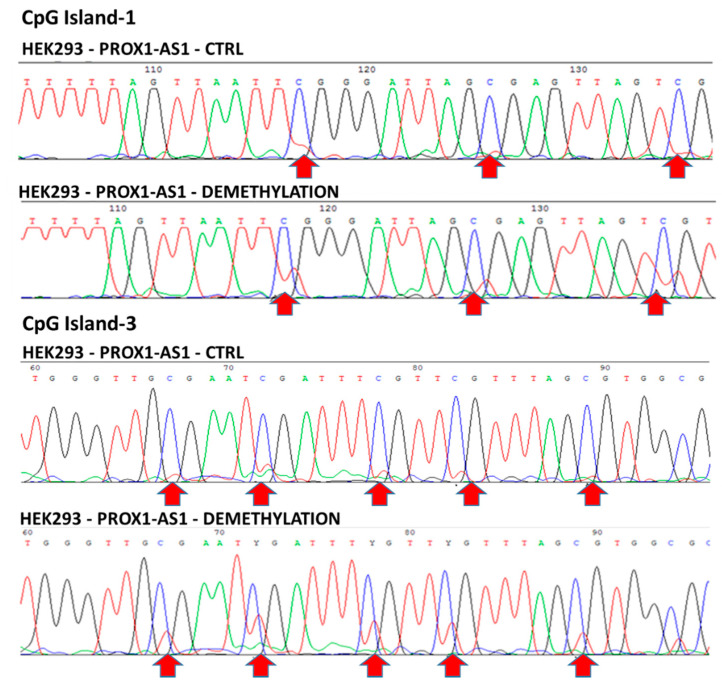
CpG-islands 1 and 3 methylation in HEK293 before and after 5-aza-2′-deoxycytidine treatment. CpG island 1 and CpG island 3 before (top line) and after (bottom line) HEK293 treatment with 5-aza-2′-deoxycytidine. Arrows indicate the location of the demethylated cytosines after the treatment with the demethylating agent (the appearance of red peaks in the bottom line corresponds to cytosine demethylation).

In particular, cytosine demethylation in CG dinucleotides was detected in CpG-islands 1 and 3 in the HEK293 line, confirming the hypothesis of a relationship between cytosine demethylation and overexpression of *PROX-AS1* (Figure 5). The increase of *PROX1-AS1* expression in RCC-derived cells (796-P and A498) after the same treatment could indicate that this effect was due to other phenomena (i.e., transcription factors, other non-coding RNA, etc.).

**Figure 6:**
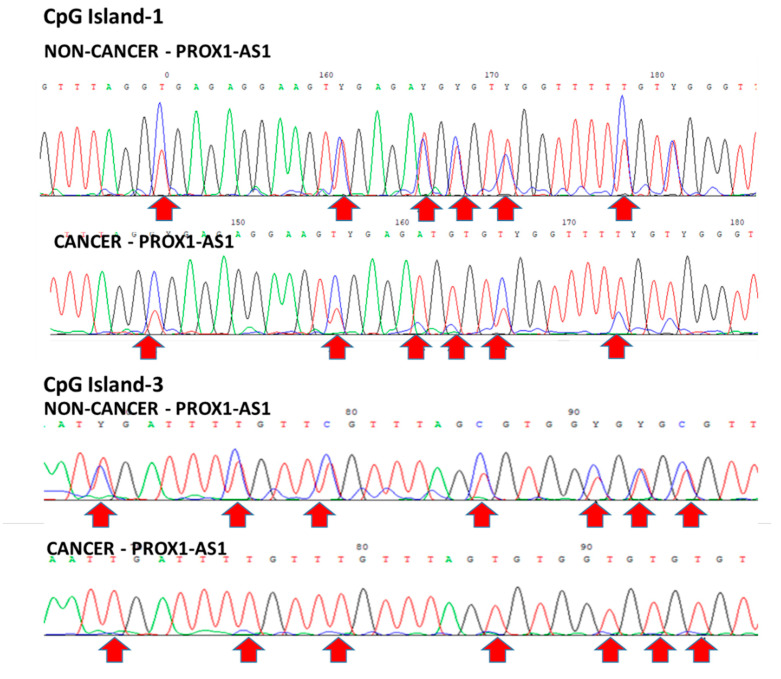
Bisulfite sequencing of paired non-tumor and tumor tissues. CpG island 1: Top-line—hemimethylation in NT (blue and red peaks). Bottom line—differential methylation (blue or red peaks) in some CG in T. CpG island 3—hemimethylation in NT (blue and red peaks) and demethylation (only red peaks) in T.

To investigate the methylation status of all the three islets of the *PROX1-AS1* gene in patient tumor tissues, we performed bisulfite sequencing of nine paired T and NT tissues. We detected CG dinucleotide hemimethylated in normal non-tumor tissues. On the other hand, in some tumor samples, complete CpG-island 3 demethylation and partial CpG-island 1 demethylation were observed (Figure 6). In the other tumor samples, no demethylation was detected for CpG-islands 1 and 3. These results support our observation of decreased expression of *PROX1-AS1* in control samples and variable higher expression in tumor specimens.

## 4. Discussion

RCC is one of the most common malignant urogenital tumors with a high metastatic behavior. Tyrosine kinase inhibitors represent the first-line treatment for this disease even though drug resistance phenomena often occur against these therapeutic strategies [35]. Therefore, investigations regarding RCC biology may clarify the exact mechanisms underlying these processes, identify novel molecular targets and valuable diagnostic markers [36].

Increasing evidence suggests that lncRNAs play crucial roles in carcinogenesis, and their dysregulation is closely related to the tumor invasion process [37,38]. Recent studies have shown that lncRNAs may be targeted for tackling RCC growth and metastasis [39]. The dysregulation of different lncRNA expression was correlated with tumor proliferation, apoptosis, metastasis, and RCC patient outcome [14,15,17,18].

A few papers have reported the potential roles of *PROX1-AS1* in cancer progression and its higher expression was noticed in ovarian, prostate, and papillary thyroid carcinoma specimens compared to control non-tumoral samples [28,32,33]. In vitro, *PROX1-AS1* overexpression or silencing regulated the proliferation, migration, and invasion of different cancer cells. To provide more insights into the role of *PROX1-AS1* in RCC, we correlated its expression in patient tumor samples with clinical data. This approach was justified by recent studies based on the coding gene—*PROX1*—and its regulatory role in RCC development and lymph node spreading [28].

In our analysis, the *PROX1-AS1* expression in tumor tissues compared to healthy control samples derived from the paired cancer lesion burden varied between kidney cancer subtypes. However, the differences were insignificant. Additionally, the observed *PROX1-AS1* increase in KIRP and AML controls can indicate a tissue-dependent expression manner.

The more important observation was the positive correlation (evaluated by Spearman’s rank-order correlation) between *PROX1-AS1* expression in cancer and non-cancer tissues in the entire patient cohort analyzed and in the KIRC subtype. Its expression increase, observed in both tissues, may result from the growing cancer lesion influence on the surrounding healthy tissue. It is worth mentioning that the tumor margin is morphologically unchanged, but at the molecular level, it is affected by the growing lesion [40,41].

We observed differences in *PROX1-AS1* expression level among gender and age groups; however, this was determined to not be statistically significant. Interestingly, *PROX-AS1* expression in tumor and non-tumoral samples among gender showed very different trends between women and men, and this phenomenon could be caused by the differential hormonal regulation on long non-coding RNA expression [42,43].

*PROX1-AS1* expression was significantly increased in patients with lymph node tumor infiltration and distant metastasis. In addition, compared to pT1 tumor stage, it increased in pT2 samples, while it decreased in pT3-pT4 samples. This bell-shaped distribution may be attributed to necrosis that occurs in higher tumor stages, affecting global measures of gene expression [44].

Furthermore, in KIRC subtype, *PROX1-AS1* expression was elevated in higher tumor grade (G3–G4 vs. G1–G2), highlighting the role of *PROX1-AS1* in tumor dissemination. This phenomenon was already observed for the coding *PROX1* gene, regulating cancer cell dissemination via lymph and angiogenesis regulation. This evidence supports the hypothesis that *PROX1-AS1* acts simultaneously on the lesion and the surrounding tissue, favoring further tumor growth.

All these observations suggest that lncRNA-*PROX1-AS1* could be considered as RCC diagnostic marker, and more analysis is needed to determine its precise involvement in different RCC phenotypes and its potential role as a therapeutic target.

The genome-wide DNA methylation study in RCC identified increased global methylation in more aggressive cancers [45,46,47]. DNA methylation was shown in RCC as a potential risk factor associated with malignant transformation [48]. Furthermore, the aberrant hypermethylation correlated with the more advanced tumor stage and grade in KIRC [49]. On the other hand, cancer-associated DNA hypomethylation was as well registered, and related with the transcriptional activation of oncogenes and consequent tumor progression [50].

It was shown that lncRNAs could regulate gene expression at the transcriptional, post-transcriptional, and epigenetic levels [8,9], while epigenetic modifications, including hyper-/hypomethylation, can modulate lncRNA expression [10]. Functional analyses established that specific lncRNAs are epigenetically activated in tumors by the loss of methylation in CpG sites of their promoter region, and a few special lncRNAs are deactivated because of increased methylation [51,52]. In the context of RCC, genomic analysis of normal kidney tissues and RCC samples revealed a series of lncRNA which expression was regulated by hyper- or hypomethylation and they were associated with poor patient outcome [53,54].

For this reason, we analyzed the CpG islands in the promoter sequence detecting three CpG islands, which can mute the *PROX1-AS1* expression. To confirm the epigenetic *PROX1-AS1* regulation, we performed in vitro global demethylation. The treatment with 5-aza-2′-deoxycytidine significantly enhanced *PROX1-AS1* expression in all the tested cell lines (769-P, A498, and HEK293) with the greatest change in human kidney HEK293 cells. We confirmed the hypermethylation of *PROX1-AS1* in the selected CpG-islands in HEK293, 769-P, and A498 cell lines by bisulfite sequencing. However, after treatment with 5-aza-2′-deoxycytidine, cytosine demethylation in CG dinucleotides was detected only in HEK293 cells (CpG-islands 1 and 3), proving specific demethylation effect in kidney cells. We assume that the enhanced *PROX1-AS1* expression after demethylation in tumor cell lines (769-P and A498) can be connected with other regulators, including transcription factors, non-coding RNAs, or genes activated upon global demethylation [55,56].

Next, we analyzed selected human specimens and detected (i) hemimethylated state of CG dinucleotides in all analyzed non-tumoral tissues, and (ii) complete, partial methylation or no methylation of CpG islands in RCC patients’ samples. These data can explain the lower *PROX1-AS1* expression in controls and variable higher expression in RCC tissues. Those findings are in concordance with the previous observations of non-coding RNA regulation via hyper- and hypomethylation in both renal cell carcinoma and healthy kidney tissues [53].

All these observations pointed out the occurrence of a possible lnc-*PROX1-AS1* epigenetic regulation and its active role in RCC progression.

## 5. Conclusions

In summary, the above results suggest the potential value of *PROX1-AS1* in RCC development and its possible epigenetic modifications, which can affect its expression pattern. The presented data may contribute to a better understanding of *PROX1-AS1* role in renal carcinoma development. Furthermore, our observations indicated that *PROX1-AS1* could be considered as a new diagnostic/prognostic marker and a potential molecular target, which should be investigated in further studies.

To better understand the role of *PROX1-AS1* in RCC progression, it is necessary to investigate the molecular mechanisms by which *PROX1-AS1* regulates RCC malignant behavior in vitro and in vivo.

## Figures and Tables

**Table 1:** Long non-coding RNAs and their implications in renal cell carcinoma.

lncRNA	Expression in Renal Tumor	Role in Renal Tumor	Mechanism	Reference
*FILNC1*	Downregulated	-Inhibition of energy metabolism, -Suppression of tumor development	Downregulation of tyrosine-protein kinase Met (c-Myc) protein	[14]
*TCL6*	Downregulated	-Sensitization of clear cell renal carcinoma to paclitaxel-induced apoptosis	Downregulation of miR-221	[16]
*lnc-DILC*	Downregulated	-Inhibition of proliferation, migration and invasion of clear cell renal carcinoma cells	Binding with phosphatase and tensin homolog (PTEN) and suppression of its degradation	[17]
*MRCCAT1*	Upregulated	-Promotion of proliferation, invasion and metastasis	Inhibition of natriuretic peptide receptor 3 (NPR3) and activation of p38-MAPK signaling	[15]
*URRCC*	Upregulated	-Enhancement of proliferation and metastasis ofclear cell renal carcinoma cells	Positive feed-forward loop with EGF like domain multiple 7 (EGFL7)/phosphor-serine/threonine-specific protein kinase (P-AKT)/forkhead box O3 (FOXO3) signaling	[18]
*MALAT1*	Upregulated	-Promotion of proliferation and invasion, -Inhibition of apoptosis, -Adverse correlation with patient survival	Interaction with enhancer of zeste 2 polycomb repressive complex 2 subunit (Ezh2) and miR-205	[19]
*lnc-ARSR*	Upregulated	-Promotion of sunitinib-resistance	Competitive binding miR-34/miR-449, facilitation of AXL receptor tyrosine kinase and c-MET expression	[20]
*lnc-TSI*	Upregulated	-Inhibitor of clear cell renal carcinoma cells metastasis	Suppression of transforming growth factor beta 1 (TGF-β) induced epithelial-mesenchymal transition	[21]

**Table 2:** The sequence of primers for bisulfite sequencing PCR.

No.	CpG-Islands (UCSC hg38)	Primers	Annealing Temperature (°C)	Fragment Length
**1**	chr1:213985384–213985737	F: GTATTTTTAGTAGGTTGAGAGGG R: CTAAATCTAACAAAAACTCCAACCC	66	233
**2**	chr1:213982658–213983508	F: TGGTATATTGGAGGAGGTATATAGG R: TACACTTCCAAACTATAACAAAAT	58	140
**3**	chr1:213979872–213980325	F: GAGGTTGTTAGGAGTTTGGTTTGTA R: AAAAAACTCTCCCACCCCCAAC	65	239

**Table 3:** Patient clinical and pathological features and *PROX1-AS1* lncRNA expression. The relative quantification value (RQ) was calculated as the relative change in *PROX1-AS1* transcript expression level compared to the *PROX1-AS1* level in the internal control represented by a mixture of RNA extracted from control samples.

Clinical and Pathological Features			Number	*PROX1-AS1* Expression in Tumor		*p*-Value	*PROX1-AS1* Expression in Non-Tumor Tissue		*p*-Value
				*Mean RQ*	*SD*		*Mean RQ*	*SD*	
**Entire Group**			42	51.9	95.6	*p* = 0.6	47.4	86.3	*p* = 0.6
**Gender**		Women	20	77.3	120.4	*p* = 0.88	37.4	79.5	*p* = 0.57
		Men	22	28.9	59.6		56.4	93.0	
**Age Group**		<45 yrs	5	37.8	77.3	*p* = 0.43	27.6	45.8	*p* = 0.9779
		≤45–65 yrs	22	73.3	115.3		45.1	81.9	
		≥65 yrs	15	25.6	59.8		57.1	104.5	
**Histopathological Type**		KIRC	33	54.3	93.4	*p* = 0.64	42.0	74.9	*p* = 0.1910
		KIRP	3	7.1	11.9		12.3	15.2	
		KICH	3	111.6	190.8		1.4	0.3	
		AML	3	11.0	10.6		187.1	166.7	
* pTNM	**Tumor Stage**	pT1	23	18.0	37.0	*p* = 0.07; (pT1 vs. pT2 *p* = 0.03)	44.8	84.8	*p* = 0.820
		pT2	5	167.1	156.1		28.1	45.7	
		pT3–pT4	14	66.6	107.9		58.4	101.9	
	**Lymph Node Invasion**	pN0	37	38.3	79.5	*p* = 0.04	50.6	90.2	*p* = 0.734
		pN1	2	215.6	55.9		57.0	73.1	
	**Metastasis**	pM0	32	32.1	64.5	*p* = 0.04	44.3	80.1	*p* = 0.578
		pM1	7	117.2	141.2		81.1	123.7	
** Grade		G1-G2	34	40.6	82.6	*p* = 0.05	46.4	88.3	*p* = 0.081
		G3-G4	5	93.3	115.3		81.6	94.5	

KIRC—clear renal cell carcinoma,—KIRP—papillary renal cell carcinoma, KICH—chromophobe renal cell carcinoma, AML—renal angiomyolipoma; type of benign renal neoplasm, * pTNM-International System of Clinico-Morphological Classification of Tumors (TNM—Tumor Node Metastasis), pT1: tumor confined to kidney > 4 cm but <7 cm, pT2: limited to kidney > 7 cm, pT3: tumor extension into major veins or perinephric tissues, but not into ipsilateral adrenal gland or beyond Gerota’s fascia, pT4: involves ipsilateral adrenal gland or invades beyond Gerota’s fascia, ** Grade: G1: Well differentiated (low grade); G2: Moderately differentiated (intermediate grade); G3: Poorly differentiated (high grade); G4: Undifferentiated (high grade).

**Table 4:** The location and characteristics of the islands.

	CpG Island-1	CpG Island-2	CpG Island-3
**Position (hg38):**	chr1:213985384	chr1:213982658	chr1:213979872
	–213985737	–213983508	–213980325
**Band:**	1q32.3	1q32.3	1q32.3
**Size (bp):**	354	851	454
**CpG count:**	40	84	42
**Percentage CpG:**	22.6%	19.7%	18.5%
**Percentage C or G**	66.7%	71.7%	70.0%

**Table 5:** CpG-islands methylation (methC) and demethylation (demethC) before and after treatment with the demethylating agent 5-aza-2′-deoxycytidine.

CpG *PROX1-AS1*	1		2		3	
	methC	demethC	methC	demethC	methC	demethC
	contr	5-AZA	contr	5-AZA	contr	5-AZA
**HEK293**	+	+	+	−	+	+
**786-P**	+	−	+	−	+	−
**A498**	+	−	+	−	+	−

## Data Availability

The data presented in this study are available on request from the corresponding author. The data is not publicly available due to privacy restrictions.
